# CD4+:CD8+ T Cell Ratio Normalization and the Development of AIDS Events in People with HIV Starting Antiretroviral Therapy

**DOI:** 10.1089/aid.2020.0106

**Published:** 2020-10-05

**Authors:** Hajra Okhai, María Jesús Vivancos-Gallego, Teresa Hill, Caroline A. Sabin

**Affiliations:** ^1^ Centre for Clinical Research, Epidemiology, Modelling and Evaluation, Institute for Global Health, University College London, London United Kingdom.; ^2^ Department of Infectious Diseases, University Hospital Ramon y Cajal and Ramon y Cajal Health Research Institute (IRYCIS), Madrid, Spain.

**Keywords:** CD4+ T cells, CD4+:CD8+ T cell ratio, antiretroviral therapy, HIV

## Abstract

We identify factors associated with the normalization of the CD4+:CD8+ T cell ratio among UK Collaborative HIV Cohort study participants, and describe the association of the CD4+ and CD8+ T cell counts and the CD4+:CD8+ T cell ratio, with the risk of new AIDS events among individuals who achieve a suppressed viral load. Participants initiating combination antiretroviral therapy (cART) after 2006 with a CD4+:CD8+ T cell ratio <1, and viral suppression within 6 months were included. Cox proportional hazard models were used to examine associations with ratio normalization (ratio ≥1). Poisson regression models were used to investigate factors associated with the development of AIDS after viral load suppression. A total of 13,178 participants [median age: 37 (interquartile range: 31–44)] were followed for 75,336 person-years. Of the 4,042 (32.9%) who experienced ratio normalization, individuals with a high CD4+ T cell count [>500 vs. ≤200 cells/mm^3^, adjusted hazard ratio (95% confidence interval): 7.93 (6.97–9.01)], low CD8+ T cell count [>1,150 vs. ≤500 cells/mm^3^: 0.18 (0.16–0.21)], and low CD4+:CD8+ T cell ratio [>0.8 vs. <0.2: 12.36 (10.41–14.68)] at cART initiation were more likely to experience ratio normalization. Four hundred and nineteen people developed a new AIDS event. Most recent CD4+ T cell count [>500 vs. ≤200 cells/mm^3^: adjusted rate ratio 0.24 (0.16–0.34)] and CD4+:CD8+ T cell ratio [>0.8 vs. <0.2: 0.33 (0.21–0.52)] were independently associated with a new AIDS event. One third of study participants experienced ratio normalization after starting cART. CD4+ T cell count and CD4+:CD8+ T cell ratio are both individually associated with ratio normalization and the development of new AIDS events after cART.

## Introduction

Untreated HIV infection is characterized by a derangement of the immune system, with reductions in the CD4+ T cell count, increases in the CD8+ T cell count, and an abnormally low CD4+:CD8+ T cell ratio,^1^ as well as increased levels of inflammatory markers, with changes occurring soon after seroconversion.^2^ Treatment with combination antiretroviral therapy (cART) generally results in rapid suppression of HIV viremia and improvements in immune markers—however, in some individuals, immune markers remain abnormal, despite viral suppression. In particular, even among those who experience improvements in their CD4+ and CD8+ T cell counts, the CD4+:CD8+ T cell ratio may remain lower than would be expected in people without HIV.^3^

In adults with HIV with virological suppression, the CD4+:CD8+ T cell ratio inversely correlates with measures of innate and adaptive immune senescence.^4^ We have previously reported that the pretreatment CD4+ T cell count is strongly associated with CD4+:CD8+ T cell ratio recovery,^5^ but it is still unclear if prolonged treatment with antiretroviral therapy (ART) will ultimately result in a full restoration of the immune system. Further research into the dynamics of the CD4+:CD8+ T cell ratio may help to understand the contribution of these measures to immune restoration post-ART.

In this study, we aim to identify factors associated with the normalization of the CD4+:CD8+ T cell ratio among participants in the UK Collaborative HIV Cohort (UK CHIC) study, and to describe the association of CD4+ and CD8+ T cell counts, as well as the CD4+:CD8+ T cell ratio, with the risk of new AIDS events among individuals who achieve a suppressed viral load.

## Methods

### Study participants

The UK CHIC study is an ongoing cohort of HIV-positive individuals (>16 years of age), who have accessed care at one or more of 25 HIV clinics in the United Kingdom at any time from 1996 onward. The study methods are described elsewhere.^6^ In brief, centers collect data on demographic information, ART treatment history, laboratory results, and AIDS diagnoses; the resulting dataset is submitted on an annual basis to the coordinating center. The project was approved by a Multicenter Research Ethics Committee (MREC/00/7/47) and by local ethics committees.

The analyses described here are based on data collected up to December 31, 2017. Participants were included in the analyses if their CD4+:CD8+ T cell ratio was <1 pretreatment initiation, and they were antiretroviral naive, started cART after 2006, were followed in the study for at least 6 months after starting cART, and experienced HIV viral load suppression during this period. In addition, participants were required to have at least one CD4+ and CD8+ T cell count measurement within the 6-month periods both before and after starting cART. Sensitivity analyses expanding the time period for suppression to 8 months were done to ensure our study did not favor individuals with more rapid suppression of viral load.

### Statistical analysis

Longitudinal CD4+:CD8+ T cell ratios were calculated using raw CD4+ and CD8+ T cell counts where both values were reported on the same date. Continuous variables were expressed as the median and interquartile range (IQR), and categorical variables as counts and percentages. To explore CD4+:CD8+ T cell ratio dynamics, the closest recorded ratios at 6, 12, 18, and 24 months after cART initiation were selected. Summary estimates were calculated for each time point with no restriction on availability of CD4+:CD8+ T cell ratio and were stratified by CD4+ T cell count and CD4+:CD8+ T cell ratio at treatment initiation.

Ratio normalization was defined as a value >1 based on previously published studies.^7^ Individuals with a normalized ratio within 6 months of cART initiation were excluded from analyses of factors associated with normalization—this was to ensure that the inclusion of individuals with a pre-cART ratio that was close to 1, in whom only minor fluctuations to the CD4+ and CD8+ T cell counts may result in a normalized ratio, did not reduce our ability to detect clinically important changes in the ratio.

For analyses of the time to ratio normalization, follow-up started on the date of ART initiation and was right censored on the earliest of date of ratio normalization, date of death, date of loss to follow-up, or December 31, 2017. Cox proportional hazard (PH) regression models were used to examine associations of demographic and clinical variables with this outcome. In particular, we examined associations with ethnic origin (white, black, or other), mode of HIV acquisition (sex between men, sex between men and women, or other/unknown), gender, age at treatment initiation, CD4+ and CD8+ T cell count/CD4+:CD8+ T cell ratio/HIV viral load at cART initiation, hepatitis B virus (HBV)/hepatitis C virus (HCV) status, AIDS status at treatment initiation, initial treatment regimen [regimens, including either non-nucleoside reverse transcriptase inhibitors (NNRTIs), protease inhibitors (PIs), integrase strand transfer inhibitors (INSTIs), or other combinations], regimen backbone [abacavir/lamivudine (ABC/TTC), zidovudine/lamivudine (ZDV/TTC), or tenofovir disoproxil fumarate/emtricitabine (TDF/FTC)], and calendar period of treatment initiation. As the ratio is calculated using the CD4+ T cell and CD8+ T cell counts, multivariable models were built separately for CD4+ and CD8+ T cell counts and CD4+:CD8+ T cell ratio. Variables significantly associated (*p* < .05) with ratio normalization in univariate analyses were selected for multivariable models.

We used Poisson regression models to investigate factors associated with the development of AIDS after cART. Individuals with an AIDS diagnosis before cART initiation were excluded from this analysis as we were particularly interested in the development of new AIDS events only. Follow-up for these analyses started at 6 months after ART initiation, so as to ensure time for the person to have achieved viral suppression and to exclude any AIDS event that may have been unmasked or caused by immune restoration inflammatory syndrome, and ended at the earliest of the date of first recorded AIDS event, date of death, date of loss to follow-up, or December 31, 2017. We examined associations with demographic factors, mode of HIV acquisition, and clinical data both at cART initiation and as time-updated covariates (CD4+ and CD8+ T cell counts, as well as the ratio, HBV/HCV status); although all participants were required to have experienced viral suppression in the first 6 months after cART initiation, we did not censor follow-up at virological rebound—instead, analyses also included adjustment for the latest HIV viral load as a time-updated covariate. In a similar manner to the models of ratio normalization, multivariable models were developed separately to include either the CD4+ and CD8+ T cell counts or the CD4+:CD8+ T cell ratio.

## Results

### Participant characteristics

A total of 27,785 UK CHIC participants were identified to have started ART between 2006 and 2017. Individuals were excluded if they had a normalized CD4+:CD8+ T cell ratio in the 6 months before initiating cART (*n* = 1,149), had <6 months follow-up (*n* = 1,998), did not achieve viral suppression within 6 months of initiating cART (*n* = 6,626), or did not have immunological data pre-ART and post-ART initiation (*n* = 4,834). The remaining 13,178 UK CHIC participants were followed for a total of 75,336 person-years.

The majority of participants were male (78.8%), of white ethnicity (59.1%), and acquired HIV through sex with men (61.2%) (Table 1). At the time of ART initiation, participants had a median age of 37 (IQR: 31–44) years had a median CD4+ T cell count of 311 (IQR: 208–440) cells/mm^3^ and a median CD4+:CD8+ T cell ratio of 0.3 (IQR: 0.2–0.5). Most participants initiated a cART regimen that included a NNRTI (59.4%). However, since the rollout of INSTIs from 2015, these have become increasingly common as part of first-line cART regimens (11.2% of those initiating cART overall, but 48.6% of those initiating cART between 2015 and 2017).

**Table 1.** Characteristics of the UK Collaborative HIV Cohort Participants Included in the Study

	*n* (%) or median (IQR) as appropriate
Total	13,178 (100.0)
Age at UK CHIC entry (median, IQR), years	37 (31–44)
Sex, *n* (%)	
Male	10,390 (78.8)
Female	2,787 (21.2)
Ethnicity, *n* (%)	
White	7,791 (59.1)
Black	3,561 (27.0)
Other/unknown	1,826 (13.9)
Mode of HIV acquisition, *n* (%)	
Sex between men	8,063 (61.2)
Sex between men and women	4,139 (31.4)
Other/unknown	976 (7.4)
Year of cART initiation	
2006–2008	3,384 (25.7)
2009–2014	7,534 (57.2)
2015–2017	2,260 (17.1)
HIV viral load at cART initiation (copies/mL)	
<10,000	4,906 (37.2)
10,000–100,000	4,851 (36.8)
100,001–500,000	2,478 (18.8)
>500,000	525 (4.0)
Unknown	418 (3.2)
CD4+ T cell count at cART initiation (median, IQR), cells/mL	311.0 (208.0–440.0)
CD8+ T cell count at cART initiation (median, IQR), cells/mL	940.0 (670.0–1,313.0)
CD4+:CD8+ T cell ratio at cART initiation (median, IQR), cells/mL	0.3 (0.2–0.5)
cART regimen, *n* (%)	
NNRTI	7,825 (59.4)
PI	3,385 (25.7)
INSTI	1,473 (11.2)
Other	495 (3.8)
Regimen backbone at cART initiation, *n* (%)	
TDF/FTC	9,970 (75.7)
ZDV/TTC	502 (3.8)
ABC/TTC	2,301 (17.5)
Other	405 (3.1)
AIDS event before cART initiation, *n* (%)	
No	11,441 (86.8)
Yes	1,737 (13.2)
HBV at cART initiation, *n* (%)	
No/unknown	12,873 (97.7)
Yes	305 (2.3)
HCV at cART initiation, *n* (%)	
No/unknown	12,578 (95.4)
Yes	600 (4.6)

%, percentage; ABC/TTC, abacavir and lamivudine; cART, combination antiretroviral therapy; HBV, hepatitis B virus; HCV, hepatitis B virus; INSTI, integrase strand transfer inhibitors; IQR, interquartile range; *n*, number of participants; NNRTI, non-nucleoside reverse transcriptase inhibitors; PI, protease inhibitors; TDF/FTC, tenofovir disoproxil fumarate and emtricitabine; UK CHIC, UK Collaborative HIV Cohort; ZDV/TTC, zidovudine and lamivudine.

### Rate of normalization

**FIG. 1.**
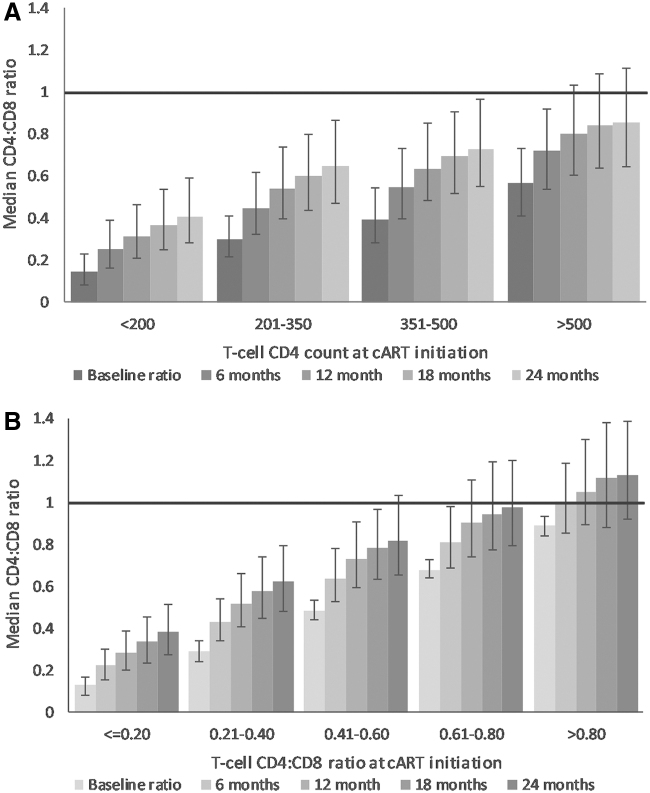
Median (IQR) CD4+:CD8+ T cell ratio at cART initiation, 6, 12, 18, and 24 months after cART initiation (*n* = 13,178), stratified by **(A)** CD4+ T cell count and **(B)** CD4+:CD8+ T cell ratio at cART initiation. Line reference at CD4+:CD8+ T cell ratio = 1. cART, combination antiretroviral treatment; IQR, interquartile range.

Figure 1 shows the median CD4+:CD8+ T cell ratio 6 monthly after starting cART, stratified by the absolute CD4+ T cell count (Fig. 1A) and the CD4+:CD8+ T cell ratio (Fig. 1B) at baseline. Over 24 months, the median CD4+:CD8+ T cell ratio doubled from 0.3 at month 0 to 0.6 at month 24. Increases were similar regardless of the absolute CD4+ T cell count or CD4+:CD8+ T cell ratio at cART initiation (0.24–0.35).

Of the 13,178 participants, 946 were excluded from further analysis as their CD4+:CD8+ T cell ratio had already normalized within the first 6 months of cART initiation. Of the remaining 12,232 participants, 4,042 (32.9%) achieved normalization over a median of 2.3 years.

Results from univariable and multivariable Cox PHs models are presented in Table 2. In univariable analyses, participants who initiated treatment in more recent years with a higher CD4+ T cell count or higher CD4+:CD8+ T cell ratio, or who initiated an INSTI-based regimen (vs. NNRTI) were more likely to experience a normalization of their ratio. In contrast, older individuals, those acquiring HIV through heterosexual exposure (vs. sex between men), those of black ethnicity (vs. white), those with an AIDS diagnosis at treatment initiation, those with HBV, and those initiating regimens, including PIs (vs. NNRTI), ABC/TTC, or ZDV/TTC (vs. TDF/FTC), were all less likely to experience normalization of their ratio. No associations were seen with sex, HCV, or HIV viral load at cART initiation.

**Table 2.** Factors Associated with Time to CD4+:CD8+ T Cell Ratio Normalization (Ratio >1), Univariable and Multivariable Cox Proportional Hazard Models (Models Were Developed Separately for the CD4+ and CD8+ T Cell Counts Themselves and the CD4+:CD8+ T Cell Ratio)

Variable	Univariable models		Multivariable models			
		*p*	(i) with absolute CD4+ and CD8+ T cell counts	*p*	(ii) with CD4+:CD8+ T cell ratio	*p*
	HR (95% CI)		aHR (95% CI)		aHR (95% CI)	
CD4+ T cell count at cART initiation (cells/mm^3^)						
≤200	1	.0001	1	.0001		
201–350	2.03 (1.85–2.23)		3.05 (2.75–3.38)			
351–550	2.42 (2.19–2.68)		4.61 (4.10–5.18)			
>500	3.45 (3.11–3.84)		7.93 (6.97–9.01)			
CD8+ T cell count at cART initiation (cells/mm^3^)						
≤500	1	.0001	1	.0001		
501–750	1.22 (1.10–1.36)		0.65 (0.58–0.73)			
751–1,150	0.93 (0.84–1.02)		0.38 (0.34–0.42)			
>1,150	0.56 (0.51–0.63)		0.18 (0.16–0.21)			
CD4+:CD8+ T cell ratio at cART initiation						
≤0.20	1	.0001			1	.0001
0.21–0.40	2.52 (2.27–2.78)				2.52 (2.27–2.79)	
0.41–0.60	5.47 (4.93–6.08)				5.69 (5.11–6.34)	
0.61–0.80	7.92 (7.01–8.96)				8.53 (7.52–9.68)	
>0.80	11.01 (9.31–13.02)				12.36 (10.41–14.68)	
Age at cART initiation/10 years older	0.89 (0.86–0.92)	.0001	0.92 (0.89–0.96)	.0001	0.93 (0.90–0.97)	.0001
Sex						
Male	1	.08				
Female	1.07 (0.99–1.15)					
Ethnicity						
White	1	.0001	1	.0001	1	.003
Black	0.77 (0.72–0.83)		0.81 (0.73–0.90)		0.85 (0.76–0.94)	
Other/unknown	0.92 (0.83–1.01)		0.88 (0.80–0.97)		0.91 (0.83–1.01)	
Mode of HIV acquisition						
Sex between men	1	.0001	1	.0001	1	.0001
Sex between men and women	0.85 (0.79–0.91)		1.23 (1.11–1.35)		1.25 (1.13–1.37)	
Other/unknown	0.89 (0.78–1.01)		0.93 (0.82–1.06)		0.91 (0.80–1.03)	
HIV viral load at cART initiation						
log_10_ copies/mL	1.01 (0.98–1.03)	.65				
AIDS event before cART						
No	1	.0001	1	.0006	1	.007
Yes	0.70 (0.63–0.77)		0.84 (0.76–0.93)		0.87 (0.79–0.96)	
HBV at cART initiation						
No	1	.002	1	.005	1	.02
Yes	0.72 (0.58–0.90)		0.73 (0.58–0.91)		0.77 (0.61–0.96)	
HCV at cART initiation						
No	1	.71				
Yes	0.97 (0.84–1.13)					
Initial cART regimen						
NNRTI	1	.0001	1	.0001	1	.002
PI	0.86 (0.80–0.92)		0.85 (0.79–0.92)		0.86 (0.80–0.93)	
INSTI	1.19 (1.05–1.34)		1.01 (0.88–1.17)		1.00 (0.87–1.14)	
Other	0.92 (0.77–1.10)		0.92 (0.74–1.14)		0.98 (0.79–1.21)	
Regimen backbone						
TDF/FTC	1	.0002	1	.0003	1	.0001
ABC/TTC	0.90 (0.82–0.97)		0.89 (0.82–0.97)		0.86 (0.79–0.94)	
ZDV/TTC	0.75 (0.64–0.89)		0.55 (0.46–0.65)		0.49 (0.41–0.58)	
Other	0.84 (0.70–1.02)		0.78 (0.62–0.98)		0.69 (0.55–0.86)	
Year of cART initiation						
2006–2008	1	.0001	1	.001	1	.0001
2009–2014	1.11 (1.04–1.19)		0.87 (0.81–0.94)		0.83 (0.77–0.89)	
2015–2017	1.44 (1.28–1.61)		0.92 (0.80–1.05)		0.83 (0.72–0.95)	

95% CIs, 95% confidence intervals; aHR, adjusted hazard ratio; HR: hazard ratio.

Similar results were seen in the multivariable models (Table 2), with those who were older at cART initiation, those of a black, other/unknown ethnic group (vs. white), and those who initiated a PI-based regimen (vs. NNRTI) or one including either ABC/TTC or ZDV/TTC (vs. TDF/FTC) being less likely to experience normalization of their ratio. In addition, individuals who had AIDS, HCV infection, or a high CD8+ T cell count at treatment initiation were also less likely to experience normalization of their ratio. Participants with higher CD4+ T cell counts at treatment initiation were more likely to experience ratio normalization. In contrast to univariable models, individuals acquiring HIV through heterosexual contact had a higher likelihood of experiencing ratio normalization, after adjusting for confounders. Sensitivity analyses removing participants with a baseline CD4+:CD8+ T cell ratio of >0.8 showed similar results.

### Risk of a new AIDS event according to the latest CD4+ and CD8+ T cell counts and the CD4+:CD8+ T cell ratio

A total of 1,737 participants who already had an AIDS event before cART initiation were excluded from these analyses. Among the remaining 11,442 people followed for 60,440 person-years, 419 people developed at least one new AIDS event after cART initiation. The majority of the participants with a new AIDS event were men (81.6%, 342/419) who had sex with men (64.4%, 270/419) and of white ethnicity (61.3%, 257/419). A large proportion of the AIDS events were unspecified (21.2%, 89/419); however, of those that were, herpes simplex disease was the most reported AIDS event (52.4%, 173/330), followed by *Mycobacterium tuberculosis* complex (14.6%, 48/330) and Kaposi's sarcoma (7.3%, 24/330).

Univariable analyses (not shown) suggested that older age, sex, ethnicity, mode of acquisition of HIV, HCV infection, HIV viral load, CD4+ T cell count, and CD4:CD8+ T cell ratio were associated with the development of a new AIDS event. Multivariable models (Table 3) suggested that, while there was no association between the CD4+ T cell count or CD4:CD8+ T cell ratio at the time of ART initiation and the development of a new AIDS event, both measures were associated with a new AIDS event when assessed over follow-up in a time-updated manner. No association was seen between the CD8+ T cell count and the development of a new AIDS event, whether considered a fixed (at ART initiation) or time-updated (over time) covariate. Analyses were repeated, expanding the period of viral suppression to 8 months, with similar conclusions.

**Table 3.** Relative Rate of AIDS Events; Results Shown Are from Four Multivariable Poisson Models

Variable	A. Covariates at treatment initiation		B. Latest covariates over follow-up	
	RR (95% CI)	*p*	RR (95% CI)	*p*
Model 1				
CD4+ T cell count (cells/mm^3^)				
≤200	1	.14	1	.0001
201–350	0.79 (0.61–1.02)		0.38 (0.25–0.57)	
351–500	0.71 (0.52–0.98)		0.33 (0.23–0.49)	
>500	0.89 (0.62–1.27)		0.24 (0.16–0.34)	
CD8+ T cell count (cells/mm^3^)				
≤500	1	.37	1	.16
501–750	0.93 (0.65–1.32)		1.25 (0.89–1.76)	
751–1,150	0.83 (0.60–1.17)		1.34 (0.96–1.87)	
>1,150	1.02 (0.73–1.44)		1.49 (1.04–2.12)	
Model 2				
CD4+:CD8+ T cell ratio				
≤0.20	1	.09	1	.0001
0.21–0.40	0.79 (0.62–1.00)		0.60 (0.38–0.95)	
0.41–0.60	0.66 (0.49–0.90)		0.51 (0.33–0.79)	
0.61–0.80	0.87 (0.59–1.28)		0.39 (0.24–0.61)	
>0.80	0.72 (0.41–1.28)		0.33 (0.21–0.52)	

Model 1 includes the CD4+ and CD8+ T cell counts themselves, and Model 2 includes the CD4+:CD8+ T cell ratio. For each model, estimates on the left (A) reflect the estimates from a model including covariates at treatment initiation (baseline) only, whereas estimates on the right (B) are from models including the latest (time updated) measurements.

Adjusted for age, sex, ethnicity, mode of HIV acquisition, year of treatment initiation, HBV, HCV, initial treatment regimen and HIV viral load.

## Discussion

In this observational cohort study, we found that only around a third of UK CHIC participants attaining viral suppression on cART achieved normalization of their CD4+:CD8+ T cell ratio over a median follow-up time of 6 years. A higher CD4+ T cell count, lower CD8+ T cell count, and higher CD4+:CD8+ T cell ratio at the time of initiating cART were each associated with an increased chance of achieving ratio normalization. Of those who had attained viral suppression within the first 6 months after starting cART, only 3.7% went on to develop a new clinical AIDS event. The latest CD4+ T cell count and CD4+:CD8+ T cell ratio were both individually strongly associated with the development of a new AIDS events on cART.

The proportion of individuals achieving ratio normalization in our study is similar to that reported by Tinago *et al.*^8^ in Ireland (26.3%) and Mussini *et al.*^7^ in Italy (29.4%), but is higher than that reported in a more recent study in Thailand,^9^ where only 18.6% of participants experienced normalization of their CD4+:CD8+ ratio over 5 years of follow-up. Of note, in the general population, an inverted CD4+:CD8+ T cell ratio is uncommon with only 8% of people 20–59 years of age in a Swedish cohort having a CD4+:CD8+ T cell ratio <1,^10^ although the prevalence of an inverted ratio did increase with age. One possible explanation for the higher proportion with a ratio <1 in our study compared to the Thai study may be earlier diagnosis^4^ and treatment initiation at higher CD4+ T cell counts; the pretreatment CD4+ T cell count in the Thai cohort was only 206 cells/mm^3^ compared to 311 cells/mm^3^ in the UK CHIC cohort.^11^ Another reason for this difference maybe access to better treatments, in particular, INSTIs. In our study, 11% of those initiating treatment were prescribed an INSTI as a first-line drug, compared to only 2% in the Thai cohort, reflecting the earlier period of follow-up of the latter. A study from a French cohort reported that initiation of cART with an INSTI regimen was strongly associated with a faster rate of ratio normalization when compared to initiation with non-INSTI containing regimens.^12^

Despite the apparent slower ratio normalization in the Thai cohort, by 10 years of follow-up, almost 40% of the Thai cohort had experienced a normalized ratio, suggesting a cumulative beneficial effect of prolonged cART exposure and viral suppression. A study comparing Asian and Caucasian people with HIV (PWH) by Petoumenos *et al.* in 2017 confirmed that the difference in the rate of ratio normalization is not explained by a difference in ability to achieve normalization *per se*, but by the lower CD4+ and CD8+ T cell counts at treatment initiation in Asian populations.^13^ In our study, those initiating treatment at lower CD4+ T cell counts or with lower CD4+:CD8+ T cell ratios still experienced improvements in their ratio over time. Importantly, the increase in CD4+:CD8+ T cell ratio is often believed to be due to the expansion of CD4+ T cells following viral suppression, with CD8+ T cells remaining abnormal.^14^ Further research into the imbalance of CD8+ T cell modulation in early HIV infection and persistence of elevated CD8+ T cells in later infection may help to understand why the CD4+:CD8+ T cell ratio does not restore to normal levels.^15–17^

We found multiple factors associated with ratio normalization, in addition to a higher CD4+ T cell count, lower CD8+ T cell count, and/or higher CD4+:CD8+ T cell ratio at treatment initiation. A diagnosis of an AIDS event before treatment initiation was associated with a reduced chance of ratio normalization, as also reported by others,^18^ likely reflecting the fact that individuals with clinical AIDS may have experienced additional immunological deficits, not captured through the T cell counts themselves, before treatment initiation. CD4+ T cell subsets are substantially affected in early infection.^19–21^ Although CD4+ T cell counts are observed to increase with initiation of cART and viral suppression, even at later stages of disease, the loss of balance in CD4+ T cell subsets during early infection may result in persistent immune activation and dysfunction.^22,23^

Those of black ethnicity were less likely to experience ratio normalization. This association has not previously been reported, but may reflect long-term immunological effects of late diagnoses and advanced disease, not adequately captured through the T cell counts. However, we did not account for continual treatment adherence after initial viral suppression in these analyses, and this may therefore be another explanation for these findings.

HBV infection before treatment initiation was associated with a lower chance of ratio normalization in multivariable models. This is similar to what has been reported previously from UK CHIC,^5^ although this association has not been seen in other cohort studies.

In line with other studies,^23,24^ we observed higher rates of ratio normalization with initiation of treatment using an INSTI in univariable analyses, although this association did not remain significant after adjustment. Our findings are likely to be limited by the relatively short follow-up time available in the era of treatment with INSTIs. Those receiving cART regimens, including TDF/FTC, were most likely to experience ratio normalization in comparison to those receiving regimens, including ABC/TTC or ZDV/TTC.

Our study also found strong associations between both the most recent CD4+ T cell count and the CD4+:CD8+ T cell ratio and the development of a new AIDS event in both univariable and multivariable models. In contrast, we found no association between these measures when assessed at cART initiation (fixed covariate analysis), suggesting these cART-related changes to these values overwhelm any longer-term prognostic value of pre-cART values. These findings are similar to those reported by Mussini *et al.*^7^ and more recently by Han *et al.*,^9^ thus highlighting the importance of clinically reviewing the CD4+ T cell count and CD4+:CD8+ T cell ratio as important clinical biomarkers.^24^

Some limitations of our study include a lack of detailed information on non-AIDS events, which meant that we were unable to assess the association between the CD4+:CD8+ T cell ratio and non-AIDS events. Reported findings on this association are conflicting.^4,7,9,25–27^ Furthermore, our analyses may be restricted by the lack of information on several unmeasured confounders in UK CHIC known to modulate immune responses, including infection by cytomegalovirus and other human herpesvirus, tobacco, and alcohol use.^28–32^ Finally, the small number of new AIDS events in the study means that our analyses may be underpowered to detect some associations.

## Conclusion

This study provides an insight into the associations of CD4+ T cell count and CD4+:CD8+ T cell ratio with ratio normalization and the development of a new AIDS event among PWH who achieved viral suppression on cART. As CD4+/CD8+ T cell counts and the CD4+:CD8+ T cell ratio were associated with ratio normalization, further research into the dynamics of T cell subsets, particularly CD8+ T cells, may help to understand why this is. It is important to continually assess CD4+ T cell count and CD4+:CD8+ T cell ratio due to their close relationship with the progression of HIV infection.

## Steering Committee

Jonathan Ainsworth, Sris Allan, Jane Anderson, Ade Apoola, David Chadwick, Duncan Churchill, Valerie Delpech, David Dunn, Ian Fairley, Ashini Fox, Richard Gilson, Mark Gompels, Phillip Hay, Rajesh Hembrom, Teresa Hill, Margaret Johnson, Sophie Jose, Stephen Kegg, Clifford Leen, Dushyant Mital, Mark Nelson, Hajra Okhai, Chloe Orkin, Adrian Palfreeman, Andrew Phillips, Deenan Pillay, Ashley Price, Frank Post, Jillian Pritchard, Caroline Sabin, Achim Schwenk, Anjum Tariq, Roy Trevelion, Andy Ustianowski, and John Walsh.

## Central Coordination

*University College London* (David Dunn, Teresa Hill, Hajra Okhai, Andrew Phillips, and Caroline Sabin) and *Medical Research Council Clinical Trials Unit at UCL (MRC CTU at UCL), London* (Nadine van Looy and Keith Fairbrother).

## Participating Centers

*Barts Health NHS Trust, London* (Chloe Orkin, Janet Lynch, and James Hand); *Brighton and Sussex University Hospitals NHS Trust* (Duncan Churchill, Stuart Tilbury, Elaney Youssef, and Duncan Churchill); *Chelsea and Westminster Hospital NHS Foundation Trust, London* (Mark Nelson, Richard Daly, David Asboe, and Sundhiya Mandalia); *Homerton University Hospital NHS Trust, London* (Jane Anderson and Sajid Munshi); *King's College Hospital NHS Foundation Trust, London* (Frank Post, Ade Adefisan, Chris Taylor, Zachary Gleisner, Fowzia Ibrahim, and Lucy Campbell); Middlesbrough, South Tees Hospitals NHS Foundation Trust, (David Chadwick and Kirsty Baillie); *Mortimer Market Centre, University College London* (Richard Gilson and Ian Williams); *North Middlesex University Hospital NHS Trust, London* (Jonathan Ainsworth, Achim Schwenk, Sheila Miller, and Chris Wood); *Royal Free NHS Foundation Trust/University College London* (Margaret Johnson, Mike Youle, Fiona Lampe, Colette Smith, Rob Tsintas, Clinton Chaloner, Caroline Sabin, Andrew Phillips, Teresa Hill, and Hajra Okhai); *Imperial College Healthcare NHS Trust, London* (John Walsh, Nicky Mackie, Alan Winston, Jonathan Weber, Farhan Ramzan, and Mark Carder); *The Lothian University Hospitals NHS Trust, Edinburgh* (Clifford Leen, Andrew Kerr, David Wilks, and Sheila Morris); *North Bristol NHS Trust* (Mark Gompels and Sue Allan); *Leicester, University Hospitals of Leicester NHS Trust* (Adrian Palfreeman and Adam Lewszuk); *Woolwich, Lewisham and Greenwich NHS Trust* (Stephen Kegg, Victoria Ogunbiyi, and Sue Mitchell), *St. George's Healthcare NHS Trust* (Phillip Hay, Christopher Hunt, Olanike Okolo, and Benjamin Watts); *York Teaching Hospital NHS Foundation Trust* (Ian Fairley, Sarah Russell-Sharpe, and Olatunde Fagbayimu); *Coventry, University Hospitals Coventry and Warwickshire NHS Trust* (Sris Allan and Debra Brain); *Wolverhampton, The Royal Wolverhampton Hospitals NHS Trust* (Anjum Tariq, Liz Radford, and Sarah Milgate); *Chertsey, Ashford and St.Peter's Hospitals NHS Foundation Trust* (Jillian Pritchard, Shirley Cumming, and Claire Atkinson); *Milton Keynes Hospital NHS Foundation Trust* (Dushyant Mital, Annie Rose, and Jeanette Smith); *The Pennine Acute Hospitals NHS Trust* (Andy Ustianowski, Cynthia Murphy, and Ilise Gunder); *Nottingham University Hospitals NHS Trust* (Ashini Fox, Howard Gees, Gemma Squires, and Laura Anderson), *Kent Community Health NHS Foundation Trust* (Rajesh Hembrom, Serena Mansfield, Lee Tomlinson, Christine LeHegerat, Roberta Box, Tom Hatton, and Doreen Herbert), *The Newcastle upon Tyne Hospitals NHS Foundation Trust* (Ashley Price, Ian McVittie, Victoria Murtha, and Laura Shewan); *Derby Teaching Hospitals NHS Foundation Trust* (Ade Apoola, Zak Connan, Luke Gregory, Kathleen Holding, Victoria Chester, Trusha Mistry, and Catherine Gatford); *Public Health England, London* (Valerie Delpech); and *i-Base* (Roy Trevelion).
